# Effect of a probiotic mixture with lactic acid activity on productive and allometric indicators in broiler chickens

**DOI:** 10.14202/vetworld.2024.1490-1496

**Published:** 2024-07-10

**Authors:** Sandra Paola Rodríguez González, Luis Miguel Borras Sandoval, Torres Vidales Giovanny

**Affiliations:** 1 Veterinary Medicine and Zootechnics Program, Pedagogical and Technological University of Colombia, Tunja, Boyacá, Colombia; 2 GIBNA; Research Group in Biochemistry and Animal Nutrition, Tunja, Boyacá, Colombia; 3 GIDIMEVEZ Research Group in Veterinary Medicine and Zootechnics, Tunja, Boyacá, Colombia

**Keywords:** broilers, lactic acid bacteria, probiotic mixture, productive indicators

## Abstract

**Background and Aim:**

The feeding and sanitary conditions significantly influence the productivity of farm animals. This study aimed to assess the impact of a lactic acid-producing microbial additive on broiler chicken productivity.

**Materials and Methods:**

A 42-day experimental period utilized 120 1-day-old Cobb 500 chicks with an average weight of 46 g. In groups of 30 each, the chicks were randomly assigned to four experimental designs. The following treatments were assessed: T1 without intervention (control), T2 with bacitracin at a concentration of 0.5 g/L, T3 with a 5% probiotic mixture (PM), and T4 with a 7.5% PM. The birds were fed the commercial balanced feed without anticoccidials daily, while vaccines were administered according to the recommended biosecurity plan by the commercial house. Drinking water was treated with PM containing lactobacilli, yeasts, and short-chain organic acids.

**Result:**

In T4, a 7.5% PM resulted in a final weight of 2361.2 g (p < 0.05), a total weight gain of 1412.8 g (p < 0.05), and improved feed efficiency with a feed conversion of 2.00 (p < 0.05), during which feed intake was lower than in the other groups.

**Conclusion:**

Microbial additives with lactic acid activity are a cost-effective and feasible solution for broiler chicken productivity.

## Introduction

Poultry farming in Colombia contributes 36.5% of the gross domestic product (GDP) for livestock; thereby, it represents 14.3% of the agricultural GDP; participating with 0.7% of the National GDP. These numbers demonstrate the important economic value and enormous potential of the poultry sector in the development of the country and its economic strength [1]. The National Federation of Poultry Farmers (FENAVI) reports for August 2022 a national broiler production of 149,606 tons and 1337 million eggs [2].

During the broiler’s 1^st^ week of life, which accounts for 16% of the productive stage, the critical transition period from yolk sac absorption to the administration of pellet food [3]. The thermoregulatory system, immunological competence, and growth patterns of the digestive system undergo maturational changes at this stage [4]. The liver, pancreas, ventricle, proventriculus, and intestines exhibit an exceptionally rapid development, about 4 times quicker than body weight growth, leading to their high efficiency [5]. An increase in the number and length of intestinal villi is vital for nutrient absorption and utilization [6].

Antibiotics were introduced into animal diets toward the end of the last century to boost meat, egg, and milk production [7]. The productive results were highly positive. This practice has drawn criticism for its potential to foster antimicrobial resistance. In 2003, the European Union banned antibiotic usage as a growth promoter [8]. In poultry production, the utilization of antimicrobials for disease prevention and productivity enhancement continues to be a contentious issue [9]. The impact of beneficial microorganisms, as emphasized by several researchers [10, 11], includes their antimicrobial, immunological, and digestive benefits. The mechanism of action of lactic acid bacteria at the intestinal level lies in the removal or elimination of enteric pathogens through the production of substances with antimicrobial activity, the suppression of toxins production by inhibiting the metabolic activity of the bacteria that produce them, and the stimulation of defense mechanisms and non-specific immunity [12, 13].

Lactobacilli, yeasts, and short-chain organic acids make up microbial additives, which are typically produced in a liquid fermentation process with a low pH. Their activity is oriented to (i) control the development of pathogenic microorganisms such as *Escherichia coli*, (ii) decrease the incidence of diarrhea, (iii) increase energy and nitrogen retention, and (iv) allow a higher weight gain [14].

Acidifiers enhance intestinal functioning and bacterial balance, improving daily feed uptake, and reducing mortality during production [15]. Organic acids such as citric, propionic, fumaric, and formic enhance gastric proteolysis and protein/amino acid digestibility [16]. This study aimed to assess the influence of a lactic acid-active probiotic mixture (PM) on broiler chickens’ productive and allometric parameters.

## Materials and Methods

### Ethical approval

All experimental procedures were performed according to the guidelines proposed by “The International Guiding Principles for Biomedical Research Involving Animals” (CIOMS, 2012). This study was approved by the Ethics Committee of the Universidad Pedagógica y Tecnológica de Colombia (September 15, 2022).

### Study period and location

The study was conducted from April 2022 to May 2022. Fieldwork was conducted in the city of Tunja, Department of Boyacá, Colombia, Florencia County, located at an altitude of 2720 m above sea level, with an average temperature of 12°C.

### Installation and equipment

The birds were housed in a shed of 4 m × 5 m (length × wide), with a metal structure, cement floor, and wood-chip bed. The household was divided into four compartments with feeders and drinkers. Heating control and curtain management were performed according to the needs of the birds, and the density per square meter was adjusted weekly according to their growth.

### Animal experiment

A total of 120 chicks of the Avian Cobb 500 line from a commercial hatchery (Pronavicola, Colombia), 1 day old, with an average weight of 46 g were used and distributed in four experimental groups (30 animals each, each groups had three replicates). The research period lasted for 42 days.

### Sanitary management

The facilities, curtains, feeders, and drinkers were washed, cleaned, and disinfected in preparation for the birds’ reception. Commercial pest control products were used for rodent and insect elimination, while the birds received vaccinations according to the scheme (Table-1).

**Table-1:** Vaccination schedule.

Date	Vaccine	Route	Strain
Day 4	Gumboro	Ocular	Bursine 2
Day 7	NDV+IBV*	Ocular	Lasota + H120
Day 14	Gumboro	Water	Bursine 3
Day 21	NDV+IBV*	Ocular	Lasota + H120

NDV=Newcastle virus, IBV=Infectious bronchitis virus

### Experimental diets

Birds received daily Itálcol® (Bogotá, Colombia) commercial balanced feed without anticoccidials. The composition of the diet changed throughout the productive stages (initiation, 1–21 days and completion, 22–42 days), as shown in Table-2. The PM was prepared as described by Borrás-Sandoval *et al*. [14]; the composition of which is described in Table-3.

**Table-2:** Composition of the base diets used in the experiment according to the stages.

Nutrients (%)	Initiation	Completion
Crude protein	22	19
Grease	2	2.4
Humidity	13	13
Fiber	5	5
Ash	8	8

**Table-3:** Components of the probiotic mixture with lactic acid activity.

Components	Contribution	Percentage
Molasses	Fermentable sugars	10.0
Urea	Non-protein nitrogen	0.5
Magnesium sulfate	Sulfur	0.2
Mineral premix (Bovine)	Minerals	0.5
Inoculum (yogurt)	Lactic acid bacteria	3.0
Water	System solvent	85.8

Treatments were supplied in the drinking water as follows from day 1 to day 42:

- Control treatment (T1): Water without product addition
- Treatment (T2): Zinc bacitracin (ALBAC®, Pzifer, Bogotá D.C., Colombia) at a dose of 0.5 g/L
- Treatment (T3): PM at 5%/L
- Treatment (T4): PM at 7.5%/L.

### Evaluation of the production parameters

The productive variables evaluated on days 1, 14, 28, and 42 were as follows [17]:

- Feed Conversion (FC): FC = Feed consumed/livestock weight
- Accumulated Weight Gain: Final weight initial weight/age (days).

### Allometric evaluation of organs of the digestive system

The selected birds (10 birds/day were euthanized on the days: 14, 28, and 42) were euthanized by a euthanasia-approved method, including sedation by inhalation of Nitrox® and then carbon dioxide for 3 min, as described previously by Chávez *et al*. [18]. All birds were slaughtered in the morning hours after fasting.

Each of the slaughtered birds underwent the conventional necropsy technique with subsequent removal and weighing of the following organs: Proventriculus, ventricle or gizzard, liver, pancreas, and entire small intestine. Each organ was washed externally and internally with sufficient water to remove food or excreta that could alter the net weight of the organ [19].

The following equation was used to determine the % live weight of each organ:

% live weight = (Organ weight/animal live weight) ×100

Allometric growth (AG) was measured as described by Campos *et al*. [20]:

AG = (On/Oh)/(PCn/PCh)

Where:

O = Organ weight

n = Days after birth

h = Birth weight

BW = Body weight.

### Statistical analysis

The Kolmogorov–Smirnov test and Levene’s test for homogeneity of variances were performed. The Tukey test at the 0.05 level was used to compare the means of the treatments. All statistical analyses were performed using Statistical Package for the Social Sciences version 11.0 (IBM Corp., NY, USA).

## Results

### Production parameters

No statistical significance (p > 0.05) was found for the initial weight parameter in the first stage (days 1–14 of age). In stage I, the final weight parameter and FC for treatment T4 (PM at 7.5%/L) were statistically different (p < 0.05) from those in treatments T1, T2, and T3. The FC differs significantly between T1 and T2, and T1 and T3 (p < 0.05), while there is no statistical difference between T2 and T3 (p > 0.05) (Table-4).

**Table-4:** Production indicators in broilers during stage I (1–14 days).

Indicators	Treatments				± EE *	p-value

	T1	T2	T3	T4		
Initial weight (g)	43.8^a^	42.4^a^	41.6^a^	42.1^a^	0.90	p > 0.05
Final weight (g)	380.9^a^	383.4^a^	389.1^a^	399.5^c^	3.813	p < 0.05
Weight gain (g)	337.1^a^	341^ab^	347.5^bc^	357.4^c^	4.180	p < 0.05
Feed conversion	1.35^c^	1.33^b^	1.31^b^	1.27^a^	0.008	p < 0.05

Control treatment (T1): Water without the addition of products. Treatment (T2): zinc bacitracin (ALBAC®, Pzifer, Bogotá D.C., Colombia) at a dose of 0.5 g/L. Treatment (T3): probiotic mixture (PM) at 5%/L. Treatment (T4): PM at 7.5%/L. ^a,b,c^Measures with different letters indicate a significant difference (p < 0.05).

* Standard error of the mean

Stage II (14–28 days) chicks’ productive performance is depicted in Table-5. For the final weight of the birds, statistically significant differences (p < 0.05) were observed among treatments T1, T2, and T3. No statistically significant difference (p > 0.05) was found between treatments T4 and T2 (zinc bacitracin) and T1 (water without the addition of products) for that parameter.

**Table-5:** Productive indicators in broilers for stage II (days 14–28).

Indicators	Treatments				± EE*	p-value

	T1	T2	T3	T4		
Initial weight (g)	380.9^a^	383.4^a^	389.1^b^	399.5^c^	3.813	p < 0.05
Final weight (g)	927.6^a^	939.7^b^	942.5^b^	948.4^c^	3.813	p < 0.05
Weight gain (g)	546.7^a^	556.3^bc^	553.4^b^	548.9^ab^	4.180	p < 0.05
Feed conversion	2.29^c^	2.20^a^	2.22^b^	2.25^b^	0.008	p < 0.05

Control treatment (T1): water without the addition of products. Treatment (T2): zinc bacitracin (ALBAC®, Pzifer, Bogotá D.C., Colombia) at a dose of 0.5 g/L. Treatment (T3): probiotic mixture (PM) at 5%/L. Treatment (T4): PM at 7.5%/L. ^a,b,c^Measures with different letters indicate a significant difference (p < 0.05).

* Standard error of the mean

The statistics revealed a significant difference (p < 0.05) between treatment methods for final weight and weight gain in the stage III phase (28–42 days). The FC ratio for T2 and T3 did not significantly differ (p > 0.05). The most productive results were achieved in the T4-treated birds (Table-6).

**Table-6:** Productivity indicators in broilers for stage III (days 28–42).

Indicators	Treatments				± EE*	p-value

	T1	T2	T3	T4		
Initial weight (g)	927.6^a^	939.7^b^	942.5^c^	948.4d	3.813	p < 0.05
Final weight (g)	2309^a^	2320^b^	2343.9^c^	2361.2d	3.813	p < 0.05
Weight gain (g)	1381.4^a^	1380.3^a^	1401.4^b^	1412.8^c^	4.180	p < 0.05
Feed conversion	2.06^b^	2.04^b^	2.05^b^	2.00^a^	0.008	p < 0.05

Control treatment (T1): water without the addition of products. Treatment (T2): zinc bacitracin (ALBAC®, Pzifer, Bogotá D.C., Colombia) at a dose of 0.5 g/L. Treatment (T3): probiotic mixture (PM) at 5%/L. Treatment (T4): PM at 7.5%/L. ^a,b,c^Measures with different letters indicate a significant difference (p < 0.05).

* Standard error of the mean

### Allometric parameters

The birds exhibited good health irrespective of their treatment, without any signs of life-threatening diseases. The daily feed supply was set at a level that prevented leftovers. No statistical interaction was detected between diet and slaughter day for any variable in this experiment, so no further independent analysis was needed.

In the ventricle, there was no significant difference in organ weight percentage of live weight between treatments (p > 0.05). However, for the proventriculus, there was no difference between treatments T2 and T3 (p > 0.05), but there was a significant difference between treatments T1 and T4 (p < 0.05). In the liver, T1, T2, and T3 showed statistically significant differences (p < 0.05), while treatment T4 did not differ significantly from T1 and T3 (p > 0.05). For the pancreatic weight, there was no significant difference between T1, T2, and T3 (p > =0.05), but a significant difference was noted between T4 and the other treatments (p < 0.05). There was no significant difference (p < 0.05) between treatments in the intestine (Table-7).

**Table-7:** Organ weight (%PV) of broilers consuming different treatments.

%PV	Treatments				SEM*
	
Organ	T1	T2	T3 (0.5%)	T4 (0.75%)	
Ventricle	2.001^a^	1.991^a^	1.768^a^	1.908^a^	0.053
Proventriculus	0.552^a^	0.459^c^	0.462^c^	0.500^b^	0.021
Liver	3.067^c^	3.552^a^	3.321^b^	3.187^bc^	0.103
Pancreas	0.297^b^	0.293^b^	0.292^b^	0.338^a^	0.011
Intestine	5.885^a^	5.389^a^	6.194^a^	5.897^a^	0.166

%PV=Percentage of live weight is determined by the formula,

* SEM=Standard error of the mean

No significant difference in ventricle allometric growth (AC) was found between T1 and T2 (p > 0.05). However, there was a significant difference between T3 and T4 (p < 0.05). In proventriculus, significant differences were found between T1 and T4 compared to T2 and T3 (p < 0.05), but not between T2 and T3 (p > 0.05). The liver showed no significant difference between T1 and T2 (p > 0.05), but distinguishable variations emerged for T3 and T4 between the two treatments.

The pancreatic variable differed significantly (p < 0.05) between treatments. The intestinal variable showed no significant differences (p > 0.05) between T1, T2, and T3, but T4 differed significantly (p < 0.05) from both T2 and T3 and was equivalent to T1 (Table-8).

**Table-8:** Allometric analysis (CA) of broilers consuming different treatments.

CA Organ	Treatments				SEM*

	T1	T2	T3 (0.5%)	T4 (0.75%)	
Ventricle	0.393^ab^	0.366^ab^	0.337^b^	0.407^a^	0.015
Proventriculus	2.376^c^	2.902^b^	2.997^b^	4.392^a^	0.430
Liver	1.065^b^	1.077^b^	1.145^a^	0.983^c^	0.033
Pancreas	2.906^a^	2.072^b^	1.53d	1.811^c^	0.296
Intestine	1.356^ab^	1.381^b^	1.269^b^	1.607^a^	0.072

CA=(On/Oh)/(PCn/PCh), where: O=Organ weight, n=Days after birth, h=Birth weight, BW=Body weight. h=Birth weight and BW=Body weight. (CA < 1)=Slow growth relative to body weight, (CA=1)=Proportional growth relative to body weight, (CA > 1)=Fast growth relative to body weight. ^a,b,c,d^Within the same row, means with a common superscript do not differ statistically (p < 0.05),

* SEM=Standard error of the mean

The highest and lowest values for %BW of each organ were found on day 42 and day 1, respectively (p < 0.05; Table-9). The various organs exhibited slow growth relative to body weight (AC < 1; Table-9).

**Table-9:** Organ weight (% BW) and allometric analysis (CA) of chicks that received different treatments during four time periods.

%PV CA	Days				
	
Organ	1	14	28	42	SEM*
Ventricle	3.02^a^	3.54^c^	2.96^d^	3.64^f^	0.015
	─	0.36^a^	0.49^c^	0.80^e^	0.005
Proventriculus	0.56^a^	0.64^c^	0.83^d^	0.91^f^	0.003
	─	0.50^a^	0.50^a^	0.82^e^	0.003
Liver	3.33^a^	4.21^c^	4.77^d^	5.45^f^	0.04
	─	0.54^a^	0.66^c^	0.86^e^	0.005
Pancreas	0.49^a^	0.29^c^	0.40^d^	0.42^f^	0.003
	─	0.24^a^	0.34^c^	0.54^d^	0.008
Intestine	8.95^a^	6.75^c^	10.33^d^	11.30^f^	0.027
	─	0.51^a^	0.51^a^	0.63^c^	0.004

%PV=Organ weight×100/(Average weight/bird), CA=(On/Oh)/(PCn/PCh), where: O=Organ weight, n=Days after birth, h=Weight at birth, PC=Body weight. (CA < 1)=Slow growth in relation to body weight, (CA=1)=Proportional growth in relation to body weight, (CA > 1)=Rapid growth in relation to body weight. ^a,b,c,d^within the same row means with a common superscript do not differ statistically (p < 0.05),

* SEM=Standard error of the mean

## Discussion

At a dose of 5- and 7.5%/L in drinking water, the application of PM led to significant improvements (p < 0.05) in the final weight, total weight gain, and FC in broilers throughout the three rearing stages compared to the control group and T2. T4 yielded the highest final weight (2.361 g; p < 0.05). The results are consistent with those reported earlier [21] using a PM of 0.5 mL/L *Bacillus subtilis* and 0.5 mL/L *Lactobacillus salivarius* in water. The lactic acid microorganisms in the PM may be responsible for the favorable reaction. Although the populations of microorganisms in the PM were not determined due to the characteristics of the inputs and the preparation conditions, the presence of lactic acid bacteria, especially *Lactobacillus*, *Streptococcus*, and *Bifidobacterium*, as well as yeasts of the Saccharomyces genus, can be deduced, as indicated in the existing literature [22, 23].

Previous studies by Attia *et al*. [24] and Duangnumsawang *et al*. [25] support the role of probiotics in enhancing gastrointestinal microbial balance, hindering pathogenic bacteria development, and bolstering the immune response. They stimulate the productions of enzymes which are responsible for enhancing digestion and absorption, leading to improved productivity. According to Rouissi *et al*. [26], while yeasts do not colonize the digestive tract, they can promote the activity of disaccharidases, stimulate the innate immune response, and produce antagonistic effects against pathogens, ultimately enhancing productive yields [27]. In addition, the presence of organic acids, mainly lactic acid, could lower the pH in the intestine, which successively inhibits the growth of pathogenic microorganisms [11].

Feed intake did not show significant variations among the experimental groups during the three experimental stages, with cumulative values ranging from 4.518 g at T4 to 4.561 g at T3. These results are similar to those reported by Emili Vinolya *et al*. [28] in broilers with two levels of enramycin (5 and 10 ppm) and tylosin phosphate (55 ppm) with values of 4.476, 4.578, and 4.580 g, respectively. In the study conducted by Park *et al*. [29], a PM was used for broiler chicken with three inclusion levels of *Bacillus subtilis*, and an increase in food consumption was observed (6,100 g) [30]. These consumption differences could be due to the fact that these authors surveyed up to day 49, while the present study collected data for up to 42 days.

In the first stage, T4 exhibited the highest FC (p < 0.05) at 1.25, followed by a decline to 2.25 in the second stage, and a recovery to 2.0 in the finishing stage. The findings agree with Moharreri *et al*. [31] in broilers provided with 0.5 mL/L Enterogermina in their drinking water. The PM dose effectively promoted beneficial bacteria growth to oppress harmful bacteria and boost nutrient absorption.

Milián *et al*. [32] affirm that these microorganisms play an important role in digestive processes because they favor an increase in the catalytic activity of digestive enzymes, allowing the degradation of macromolecules into smaller ones, which are easily diffused and absorbed by the intestinal walls. Likewise, Anadón *et al*. [33] pointed out that the administration of probiotics can stimulate the immune system in several ways: Generating increased macrophage activity and a greater capacity to phagocytose microorganism particles, increasing the production of immunoglobulins G and M and interferon, and increasing local antibodies on mucosal surfaces [34]. Improving birds’ sanitary status through this aspect subsequently enhances FC efficiency.

The intestine increases its RW by nearly double within the first 48 h after birth, while the small intestine specifically undergoes positive allometric growth during that time, as shown in Table-7, in accordance with [35]. Similarly, Lu *et al*. [36] reported that the RW of the small intestine decreases on day 7 of age; however, diets play an important role in the variation of allometric parameters (intestinal weight and length), which causes changes or alterations within 24 h after feeding [13]. Greater gains in weight result from an elevated liver and pancreas mass attributed to a heightened metabolic rate [37].

The pancreas, duodenum, and jejunum, weighing more significantly in birds that fed on T3 and T4, were followed by the liver and ileum. This result corresponds to previous findings [38, 39]. A larger pancreas may enhance jejunum amylase activity, improving starch digestibility [40].

Other results suggest that no differences were obtained in the RW of the pancreas [41] at the end of feed restriction, but a higher weight was obtained 1 week later and even at the end of the productive cycle; this increase responds to the greater need of enzymes for digestion, due to the higher feed consumption, as well as to the percentage of body weight, that the birds have once they are allowed and stimulated permanent access to feed, results that are evidenced by those presented in this study.

On 8 days of age, the pancreas exhibited superior allometric growth compared to the liver in chickens administered the hydrated nutritional supplement [42, 43]. On 14 and 21 days of age, the allometric growth of the small intestine varied statistically between treatments, with the most significant growth occurring in T3 and T4.

## Conclusion

Based on the yield parameters achieved, including weight gain and FC, the combination of T3 (5%/L) and T4 (7.5%/L) PM showed synergy akin to antibiotic growth promoters. In the control treatment, the allometric growth of the liver and intestine was more significant than in treatments utilizing the PM.

## Authors’ Contributions

LMBS, TVG, and SPRG: Conceived and designed the study. LMBS, TVG and SPRG: performed the study, collected and analyzed data, interpreted the data, and drafted and revised the manuscript. LMBS and TVG: Data collection and analysis. LMBS, TVG, and SPRG: Conceived the idea and drafted and edited the manuscript. All authors have read, reviewed, and approved the final manuscript.
